# NOS2 expression in glioma cell lines and glioma primary cell cultures: correlation with neurosphere generation and SOX-2 expression

**DOI:** 10.18632/oncotarget.16106

**Published:** 2017-03-10

**Authors:** Paola Palumbo, Gianfranca Miconi, Benedetta Cinque, Francesca Lombardi, Cristina La Torre, Soheila Raysi Dehcordi, Renato Galzio, Annamaria Cimini, Antonio Giordano, Maria Grazia Cifone

**Affiliations:** ^1^ Department of Life, Health and Environmental Sciences, University of L’Aquila, L’Aquila, Italy; ^2^ Department of Surgery, Operative Unit of Neurosurgery, San Salvatore Hospital, L’Aquila, Italy; ^3^ Sbarro Institute for Cancer Research and Molecular Medicine and Center for Biotechnology, Temple University, Philadelphia, PA, USA; ^4^ National Institute for Nuclear Physics (INFN), Gran Sasso National Laboratory (LNGS), Assergi, Italy; ^5^ Department of Medicine, Surgery and Neuroscience, University of Siena, Siena, Italy

**Keywords:** glioma, primary cultures, cancer stem cell, NOS2, SOX-2

## Abstract

Nitric oxide has been implicated in biology and progression of glioblastoma (GBM) being able to influence the cellular signal depending on the concentration and duration of cell exposure. NOS2 (inducible nitric oxide synthase) have been proposed as a component of molecular profile of several tumors, including glioma, one of the most aggressive primary brain tumor featuring local cancer stem cells responsible for enhanced resistance to therapies and for tumor recurrence. Here, we investigated the NOS2 mRNA expression by reverse transcription-PCR in human glioma primary cultures at several grade of malignancy and glioma stem cell (GSC) derived neurospheres. Glioma cell lines were used as positive controls both in terms of stemness marker expression that of capacity of generating neurospheres. NOS2 expression was detected at basal levels in cell lines and primary cultures and appeared significantly up-regulated in cultures kept in the specific medium for neurospheres. The immunofluorescence analysis of all cell cultures to evaluate the levels of SOX-2, a stemness marker aberrantly up-regulated in GBM, was also performed. The potential correlation between NOS2 expression and ability to generate neurospheres and between NOS2 and SOX-2 levels was also verified. The results show that the higher NOS2 expression is detected in all primary cultures able to arise neurosphere. A high and significant correlation between NOS2 expression and SOX-2 positive cells (%) in all cell cultures maintained in standard conditions has been observed. The results shed light on the potential relevance of NOS2 as a prognostic factor for glioma malignancy and recurrence.

## INTRODUCTION

Malignant gliomas are the most common type of primary intracranial tumor in adults [1]. Gliomas arise from glial cells and are classified on histologic subtypes according to World Health Organization (WHO) classification grade that organizes all gliomas to I–IV grades (I-II low grade, III-IV high grade glioma) based on malignant behavior. Gliomas are locally invasive cancers that show marked anaplasia, malignization, proliferation, invasiveness, besides, they are less sensitive to radiotherapy and are highly chemo-resistant [2–5], leading to tumor relapses after surgical exeresis.

Clinically, malignant glioma treatment includes maximal safe surgical resection, radiotherapy and chemotherapy with temozolomide (TMZ) [2, 3, 5]. Although new therapeutic strategies have been improved in last years [6], the survival rate strictly depends on glioma subtype: glioblastoma (IV grade - GBM) has the poorest overall survival, with a median survival of 15 months from diagnosis and less than 5% of patients survive 5 years post diagnosis [7].

Gliomas are characterized by the presence of glioma stem cells (GSCs), an extremely small tumorigenic cell population showing a high ability to self-renew, ability to generate non-tumorigenic cells and to reveal the potential multilineage differentiation, expression of high levels of undifferentiated stem cell markers like nestin, SOX-2 and β-tubulin III [8–11]. The ability to promote glioma relapse, angiogenesis, invasiveness and therapeutic resistance, renders GSCs a potential target for anti-glioblastoma therapy [10, 12–16]. Several studies suggest that gliomas are characterized by a markedly inflammatory environment and the inflammation seems to be involved in all steps of tumorigenesis promoting genomic instability, proliferation and survival of malignant cells, angiogenesis, resistance to therapy, local or systemic immunosuppression and also rising the metastatic process [17–20].

In particular, Salazar-Ramiro *et al*. [21] sustained that inflammation increases cancer risk leading to development and progression of cancerous cells, besides, inflammatory cells are frequently in tumors that expressed cytokines, growth factors, prostaglandins, reactive oxygen species (ROS), extracellular matrix-degrading enzymes and angiogenic factors (vascular endothelial growth factor -VEGF), and MMP9. The inflammatory enzyme Nitric Oxide Synthase 2 (NOS2) and its product nitric oxide (NO) have been implicated in the pathophysiology of several inflammatory disorders and human cancers [22, 23]. A recent all-comprehensive review reported the effects of NOS and NO on glioma cell biology [24]. Eyler *et al*. [25] reported that glioma stem cells expressed high NOS2 levels which in turn seem to be involved in their proliferation and tumor growth. Of note, NOS2 mRNA correlates with a worse glioma patient survival. Moreover, according to the same Authors, the silencing of NOS2 expression by RNA interference decreased *in vitro* brain tumor initiating cells, highlighting the main role of NOS2 in tumor stem cell biology and maintenance. Papaevangelou *et al*. [26] have reported the key role of NOS2 in tumor development and vessel maturation in C6 rat glioma cell line. Shen *et al*. [27] after treating a co-culture of U87-MG and C6 glioma cell lines in transwell plates with the NO donor sodium nitroprusside (SNP), or NAME (Nω-nitro-l-arginine methyl ester), a NOS inhibitor, recorded respectively the increase and the significant inhibition of tumor cell migration. NOS2 knockdown by RNA interference strategy or by specific inhibitors, negatively affects the proliferation and invasiveness of glioblastoma cells [28, 29] and was able to reduce the progression of subcutaneous and intracranial human glioma xenografts in mice [25]. Taken together, these findings strongly suggest that NOS2 could represent an interesting therapeutic target, so a pharmacological modulation of NOS expression could be considered in the treatment of malignant glioma.

The first aim of this study was to evaluate the NOS2 expression in human glioblastoma primary culture to verify its association with the capacity of generating neurospheres. In addition, even owing to our previous experience on the role of SOX-2 in human glioma primary cultures [9], we have also analyzed the potential correlation between NOS2 and SOX-2 in both glioma primary cultures and derived neurospheres. Glioma cell lines were used as positive controls both in terms of stemness marker expression that of capacity of generating neurospheres.

## RESULTS

### Neurosphere generation and immunofluorescence analysis of glioma cell lines

The ability of glioma cell lines, U-251 MG, T98G, U-87 MG, U-373 MG, LN229, and glioma primary cultures to generate neurospheres was evaluated in the specific medium for GSC growth. Human glioma cell lines were used as a control for stemness characterization and ability to form neurosphere, according to the available literature data [30–32].

**Figure 1:**
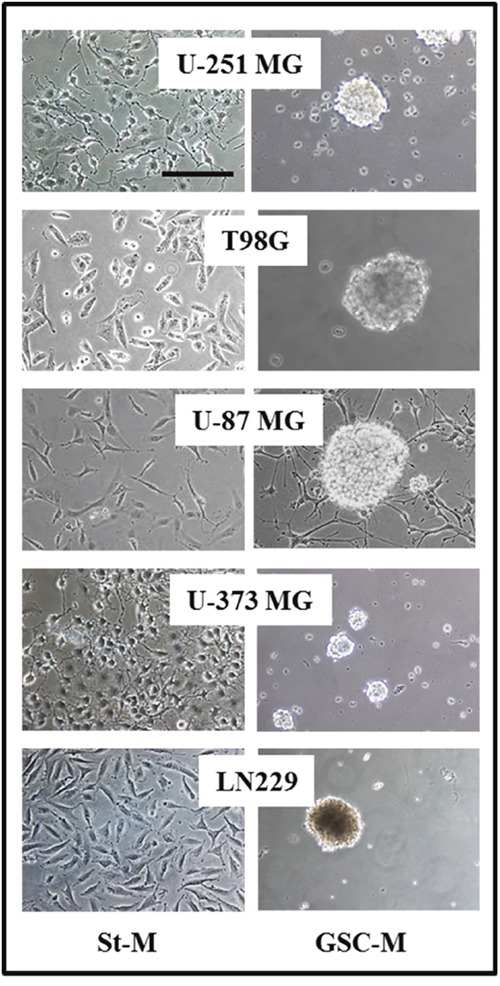
Glioma cell lines cultured in standard culture medium (St-M) and in DMEM/F12 medium serum free with EGF, b-FGF and B27 supplement for neurosphere growth (GSC-M) Representative images from the analyzed glioma cell lines are shown (Original magnification 20X, scale bar = 100 μm).

The glioma cell lines were cultured both in standard culture medium (DMEM supplemented with FBS 10% - “St-M”) and in culture medium for GSC generation (DMEM/F12 medium serum free with EGF, b-FGF and B27 supplement - “GSC-M”). Figure 1 shows the morphology of glioma cell lines U-251 MG, T98G, U-87 MG, U-373 MG, LN229 and respective neurospheres observed by contrast phase microscope. All cell lines kept in GSCs medium were able to generate *in vitro* neurospheres at different time-points. In particular, the U-87 MG cell line started to organize in neurospheres after 8-10 days, LN229 after 12-15 days, T98G after 16-18 days, U-373 MG and U-251 MG after 20-22 days.

**Figure 2:**
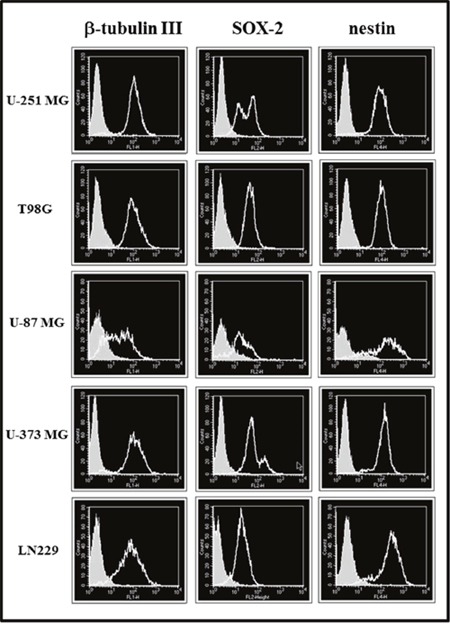
Cytofluorimetric analysis for β-tubulin III, SOX-2 and nestin expression in glioma cell lines maintained in St-M (clear profile) Full areas show the autofluorescence. The cytofluorimetric profiles from one representative of two independent experiments are shown.

**Figure 3:**
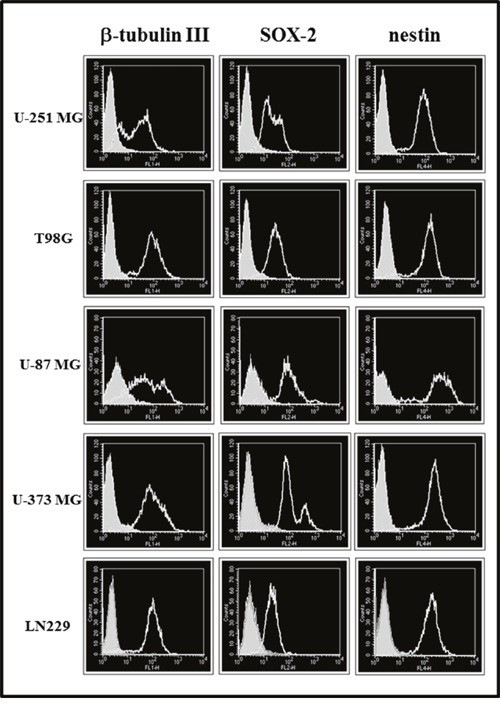
Cytofluorimetric analysis for β-tubulin III, SOX-2 and nestin expression in glioma cell lines maintained in GSC-M (clear profile) Full areas show the autofluorescence. The cytofluorimetric profiles from one representative of two independent experiments are shown.

**Figure 4:**
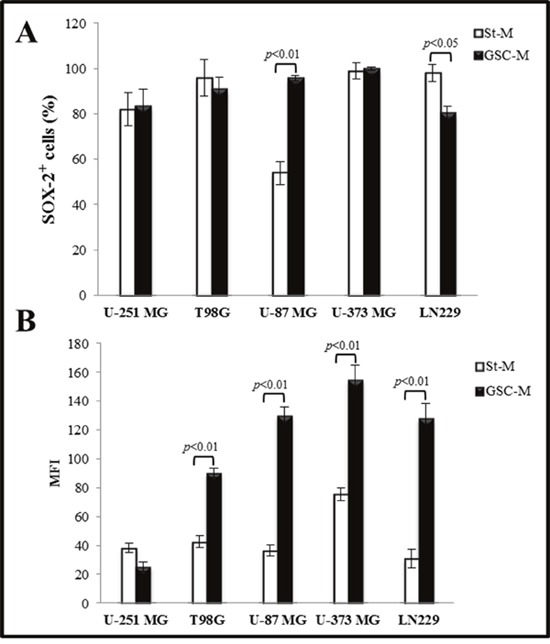
SOX-2 immunostaining analysis of glioma cell lines **(A)** Percentage (%) of SOX-2 positive cells in glioma cell lines maintained both in St-M (white bars) and GSC-M (black bars) evaluated by cytofluorimetric analysis. Data are presented as the mean values of one representative out of two independent experiments in duplicates ± SD. **(B)**Values of Median Fluorescence Intensity (MFI) of SOX-2 in glioma cell lines maintained both in St-M and GSC-M. Data are from one representative out of two independent experiments in duplicates ± SD.

Moreover, we examined the stemness markers β-tubulin III, SOX-2, and nestin by immunofluorescence analysis. The results in all cell lines cultured in St-M or GSC-M are reported in Table 1 and the representative profiles shown in Figures 2 and 3, respectively. In order to allow an easier comparison among SOX-2 expression in all glioma cell lines, Figure 4 shows the SOX-2 levels detected in St-M and in GSC-M either as % SOX-2 positive cells or Median Fluorescence Intensity (MFI) values (Figures 4A and 4B, respectively). Of interest, the mean levels of MFI in GSC-M (mean ± SEM, 105.56±14.30) resulted significantly higher (*p*<0.001) when compared to St-M (mean ± SEM, 45.77±4.72).

**Table 1:** Percentage (%) cells expressing β-tubulin III, SOX-2 and nestin in glioma cell lines maintained in standard culture conditions (St-M) and in DMEM/F12 medium serum free with EGF, b-FGF and B27 supplement for neurosphere generation (GSC-M)

	β-tubulin III		SOX-2		nestin	
	St-M	GSC-M	St-M	GSC-M	St-M	GSC-M
**U-251 MG**	97.5 ± 8.2	68.4 ± 6.1	82.0 ± 7.4	83.5 ± 7.3	98.1 ± 4.1	97.0 ± 1.5
**T98G**	98.9 ± 7.4	96.6 ± 7.2	95.8 ± 8.1	91.0 ± 5.0	99.0 ± 1.5	94.3 ± 2.7
**U-87 MG**	52.2 ± 5.0	79.0 ± 6.3	54.0 ± 5.0	95.8 ± 1.1	99.7 ± 2.4	99.0 ± 1.6
**U-373 MG**	97.0 ± 1.9	98.5 ± 3.5	98.8 ± 3.6	99.9 ± 0.8	98.6 ± 1.8	98.7 ± 2.5
**LN229**	90.1 ± 4.9	93.4 ± 4.2	98.0 ± 3.8	80.8 ± 2.1	85.4 ± 3.0	96.7 ± 1.6

(St-M = *standard medium* DMEM supplemented with FBS 10%; GSC-M= *medium for GSC generation*). Data shown are presented as the mean values of one out of two independent experiments in duplicates ± SD.

### NOS2 mRNA expression in glioma cell lines

**Figure 5:**
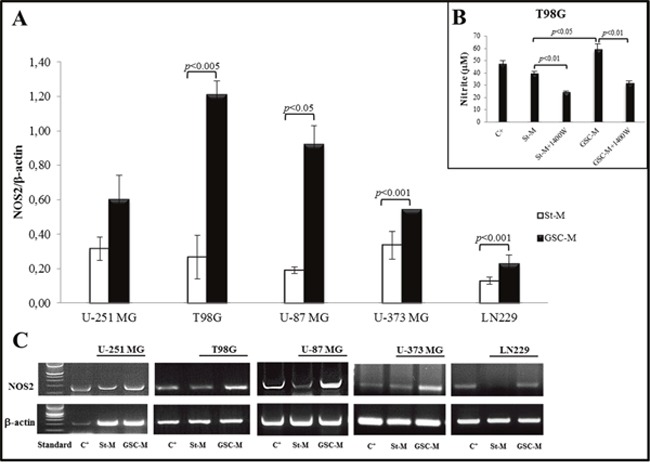
NOS2 expression in glioma cell lines and respective neurospheres **(A)** The graph shows the mean values obtained from ratio of the NOS2 expression density to endogenous control b-actin ± SEM of three independent experiments in duplicate. **(B)** Nitrite levels (μM) detected in T98G cell line kept in St-M and in GSC-M with or without NOS2 inhibitor, 1400W (100 μM) for 24 hrs. *C^+^=*Positive control (human non-small cell lung cancer A549 cells treated for 24 hrs with inflammatory cytokines (IFN-γ 10 ng/ml, IL-1β 10 ng/ml and TNF-α 10 ng/ml) and LPS 100 μg/ml. Data are from one representative experiment performed in duplicate ± SD. **(C)** Images from one representative out of three independent experiments of NOS2 expression in glioma cell lines maintained in St-M and in GSC-M are shown. *Standard* = DNA ladder (100 bp), *C^+^=*Positive control (human A549 cells treated with inflammatory cytokines and LPS). β-actin was used as the internal control.

In order to determine whether glioma cell lines cultured in St-M and GSC-M expressed NOS2, a RT-PCR technique was used as above described. Densitometric analysis showed that NOS2 mRNA levels in neurosphere cultures (GSC+) from T98G, U-87 MG, and U-373 MG were significantly higher when compared to the respective cells in St-M (Figure 5A). Moreover, the electrophoretic bands revealed that glioma cell lines maintained at St-M exhibited a basal expression of the gene encoding NOS2. NOS2 expression in St-M or GSC-M cells was associated to the enzymatic activity of NOS2 as analysed by nitrite levels in the presence or absence of NOS2 inhibitor 1400W. Representative nitrite levels produced by T98G cells, kept in St-M or GSC-M conditions, after 24 hrs incubation in the presence or absence of 100 μM 1400W are reported in Figure 5B. Figure 5C shows the representative images of three independent RT-PCR experiments aimed to evaluate the NOS2 gene expression in glioma cell lines (U-251 MG, T98G, U-87 MG, U-373 MG and LN229) cultured both in St-M or in GSC-M conditions.

### Morphology of human glioma primary cultures and derived-neurospheres

**Figure 6:**
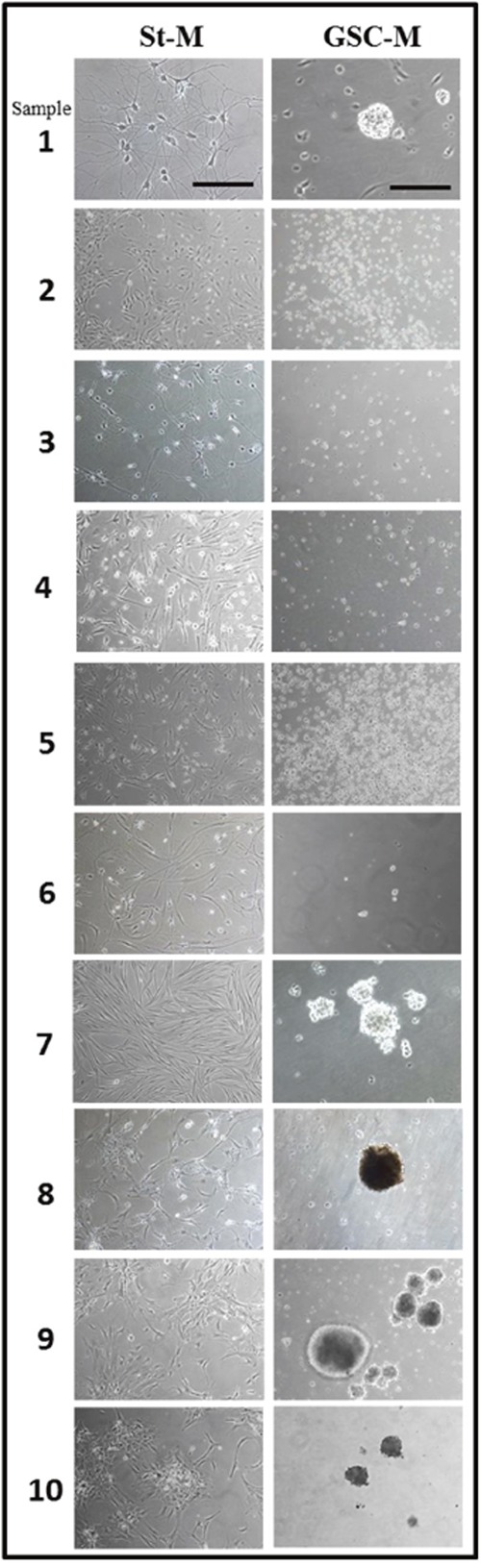
Morphology of glioma primary cultures and respective neurospheres when generated Representative phase contrast images of primary glioma cell culture maintained in St-M (10X magnification, scale bar = 200 μm) and in GSC-M condition (20X magnification, scale bar = 100 μm).

All the post-surgical glioma specimens at different malignancy (6 biopsies of IV grade, 1 of III grade and 3 of low grade glioma) were enzymatically digested and obtained cells were cultured both in St-M and in GSC-M. The ability to generate neurosphere (GSC+) or not (GSC-) was *in vitro* evaluated by microscopic observation. In Figure 6 representative images from all the 10 glioma primary cultures acquired in standard conditions (St-M) and in GSC-M are shown. 5 out from 10 cultures were able to generate neurospheres (GSC+), in particular 4 of IV grade and 1 of Low grade glioma (samples #1, #7, #8, #9 and #10). A morphological heterogeneity was observed in St-M primary cultures: astrocytic-shaped cells with long cellular processes (samples #1 and #3), fibroblastic-shaped cells (samples #4, #5, #6, #7), epithelioid-like cells and spindle-shaped cells (samples #2, #8, #9, #10). A morphological variability was also detected in neurophere cultures: spheres of different sizes were observed, some of them with a well-defined spherical shape (samples #1, #8, #9), or showing irregular cell clusters (sample #7 and #10). The results of cytofluorimetric analysis for detection of β-tubulin, SOX-2 and nestin markers in all primary cultures and in the relative neurospheres are reported in Table 2. Confirming previous findings [9], in all primary cultures β-tubulin and nestin positive cell percentages were high in both culture conditions. On the other hand, the levels of SOX-2, which appeared overexpressed in all cultures maintained in GSC-M, were rather heterogeneous in cells kept in St-M.

**Table 2:** Percentage (%) cells expressing β-tubulin III, SOX-2 and nestin in glioma primary cultures maintained in standard culture conditions (St-M) and in DMEM/F12 medium serum free with EGF, b-FGF and B27 supplement for neurosphere generation (GSC-M)

	β-tubulin III		SOX-2		nestin	
	St-M	GSC-M	St-M	GSC-M	St-M	GSC-M
**1**	92.35 ± 2.62	92.91 ±2.65	90.4 ± 4.52	97.84 ± 4.89	94.68 ± 3.73	95.92 ± 4.80
**2**	74.71 ± 3.74	/	14.56 ± 0.73	/	87.14 ± 2.36	/
**3**	80.1 ± 4.01	/	28.3 ± 1.42	/	79.98 ± 4.00	/
**4**	97.51 ± 4.88	/	8.01 ± 0.40	/	84.25 ± 4.21	/
**5**	38.77 ± 1.94	/	13.78 ± 0.69	/	43.25 ± 2.16	/
**6**	82.53 ± 2.13	/	35.99 ± 1.80	/	77.96 ± 3.90	/
**7**	90.77 ± 3.54	99.93 ±4.20	69.33 ± 3.47	84.01 ± 3.70	90.02 ± 4.50	99.99 ± 5.00
**8**	95.31 ± 4.77	98.86 ± 4.14	82.03 ± 4.10	84.71 ± 2.94	96.76 ± 4.84	99.35 ± 2.87
**9**	88.74 ± 1.44	94.78 ± 3.54	69.3 ± 3.47	80.30 ± 3.02	90.87 ± 3.54	99.63 ± 3.98
**10**	60.9 ± 3.05	81.30 ± 4.07	81.00 ± 3.05	65.73 ± 3.29	93.9 ± 2.70	95.40 ± 4.77

Data shown are presented as the mean values of one out of two independent experiments in duplicates ± SD.

### Analysis of NOS2 expression levels in glioma primary cultures and derived-neurospheres

**Figure 7:**
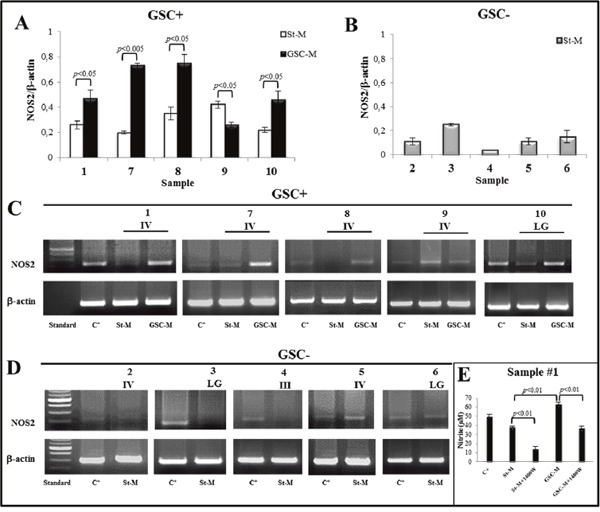
NOS2 expression and activity in primary cell cultures NOS2 mRNA expression was detected by RT-PCR in glioma primary cultures obtained from human glioma specimens at several grade of malignancy (VI, III and Low Grade (LG)), cultured in standard medium DMEM 10% FBS (St-M) and in specific medium for neurosphere generation (GSC-M). **(A)** GSC+: histograms showing the densitometric analysis of NOS2 expression in primary cultures kept in St-M (white bars) and in GSC-M (black bars). **(B)** GSC-: histograms showing the densitometric analysis of NOS2 expression in primary cultures maintained in St-M (grey bars) unable to generate neurosphere. Data are presented as mean values ± SEM of three independent experiments in duplicate. **(C)** RT-PCR of NOS2 from 5 GSC+ cultures. **(D)** RT-PCR of NOS2 from 5 GSC- cultures. Images from one representative out of three independent experiments of NOS2 expression in glioma primary cultures maintained in St-M and in GSC-M are shown. *Standard* = DNA ladder (100 bp), *C^+^=*Positive control (human non-small cell lung A549 cells treated with inflammatory cytokines and LPS). β-actin was used as the internal control to normalize the expression level of NOS2. **(E)** Nitrite concentration (μM) in primary culture #1 kept in St-M and GSC-M with or without NOS2 inhibitor, 1400W (100 μM), for 24 hrs. *C+*= Positive control (human non-small cell lung cancer A549 cells treated with inflammatory cytokines and LPS for 24 hrs). Data are from one representative experiment performed in duplicate ± SD.

**Figure 8:**
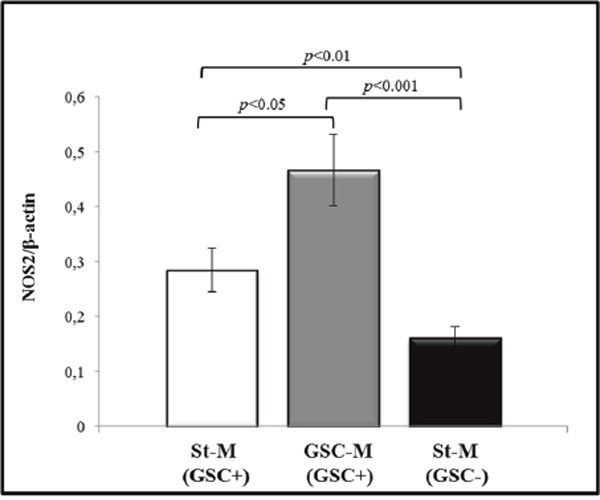
Densitometric analysis of NOS2 expression levels Analysis of NOS2 levels in the 5 primary cultures maintained in St-M and able to generate neurospheres (white bar), in the respective neurospheres (grey histogram) and in the 5 primary cultures in St-M that did not generate neurospheres (black histogram). Histogram bars represent the mean of data shown in Figure 7 ± SD.

NOS2 mRNA expression levels were evaluated in glioma primary cultures as well in their derived-neurospheres, when generated. The results from densitometric analysis of NOS2 expression in GSC+ or GSC-, as normalized to relative β-actin levels, are shown in Figure 7A and 7B, respectively. Of note, NOS2 expression levels in neurospheres from samples #1, #7, #8, and #10 were significantly higher when compared to their respective primary cultures in St-M (Figure 7A). Figure 7C and 7D show representative PCR products on agarose gels from GSC+ and GSC- cell extracts, respectively. Accordingly, the nitrite levels in GSC-M cell supernatants were significantly higher (*p*<0.01) when compared to St-M cells. The nitrite generation attributable to NOS2-dependent NO synthesis were that inhibited by 1400W. In Figure 7E the results from a representative case (sample #1) are shown. NOS2 expression levels of all GSC+ were significantly higher (*p*<0.05) when compared to their respective primary cultures maintained in St-M (Figure 8). Interestingly, the expression of NOS2 mRNA was significantly (*p*<0.01) upregulated in glioma primary cultures GSC+ at St-M respect to all cultures GSC- at St-M (Figure 8).

### SOX-2 expression in glioma primary cultures and correlation with NOS2

**Figure 9:**
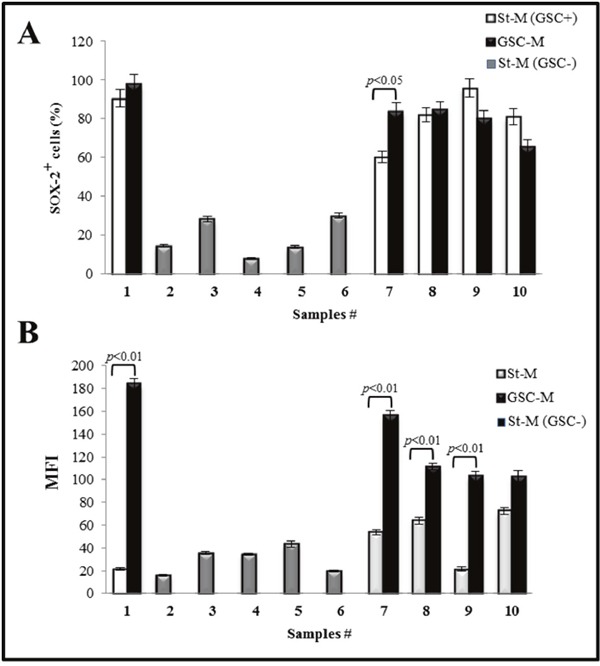
SOX-2 immunostaining analysis of glioma primary cultures **(A)** The percentage (%) of SOX-2 positive cells was acquired by flow cytometry. White bars are relative to primary cultures in St-M and able to generate neurospheres when cultured in GSC-M (GSC+) and grey bars are relative to primary cultures in St-M not able to generate neurospheres when cultured in GCS-M (GSC-). Black bars are relative to neurosphere cultures (GSC-M). Data are presented as the mean values of one representative out of two independent experiments in duplicates ± SD **(B)** Values of Median Fluorescence Intensity (MFI) of SOX-2 in glioma primary cultures maintained in St-M and in GSC-M able or not to generate neurospheres. Data are from one representative out of two independent experiments in duplicates ± SD.

SOX-2 expression was also investigated in all primary cell cultures both at St-M and GSC-M conditions. GSC- cultures (2 IV grade, 1 III grade and 2 low grade glioma) expressed low levels of SOX-2 positive cells (<30%; range: 8.0–30.02%, average value: 18.9%) (Figure 9A, grey bars). On the contrary, the St-M cultures able to generate neurospheres showed high levels of SOX-2 expressing cells (>60%; range: 60.33–95.87%, average value: 81.9%) (Figure 9A, white bars). As expected, the percentage of SOX-2 positive cells in GSC-M culture (black bars) was >60% (range: 62.80–99.6%, average value: 82.49%). In Figure 9B SOX-2 expression levels are reported as MFI values. Taken together, the results clearly show a significant increase (*p*<0.00001) in the level of SOX-2 expression in GSC-M (mean±SEM, 131.48±11.01) versus the relative St-M cells (mean±SEM, 46.78±7.18).

**Figure 10:**
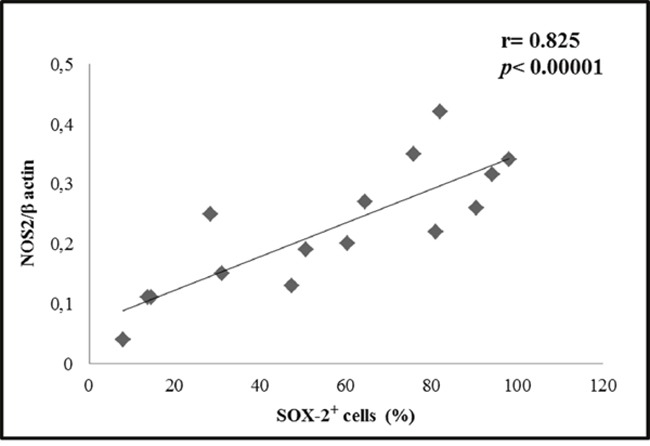
Correlation between NOS2 and SOX-2 Correlation between NOS2 level expressed as mean values of NOS2/β-actin ratio and % SOX-2 positive cells (mean values) detected in 10 primary cultures and in 5 cell lines (U-251 MG, T98G, U-87 MG, U-373 MG, LN229) kept in standard conditions (St-M) and analyzed by Pearson's test.

In order to verify a statistical relationship among NOS2 expression levels and SOX-2 positive cells across all glioma primary cultures and cell lines kept in St-M, a correlation analysis was conducted. Pearson's test revealed a positive, statistically significant and strong correlation between NOS2 expression levels and % SOX-2 positive cells (r= 0.825; *p*<0.00001) (Figure 10).

## DISCUSSION

The aim of the present work was firstly to analyse the expression level of NOS2 in human glioma primary cultures able or not to generate neurospheres. Glioma cell lines able to generate neurospheres were used as positive controls. NOS2 expression and activity, which were detected at different levels in all glioma cell lines kept in standard conditions, significantly increased in the derived-neurospheres. Similarly to cell lines, glioma primary culture-derived neurospheres expressed significantly higher levels of NOS2 expression and activity when compared to the cells cultured in standard conditions. Of note, all primary cultures from malignant gliomas unable to generate neurospheres (GSC-) expressed low levels of NOS2, highlighting its possible relevance in glioma pathology as reported by other groups [24, 25, 28, 29]. The potential correlation between NOS2 and SOX-2, a stemness marker aberrantly upregulated in GBM [33–35], was also verified. A high and significant correlation resulted between NOS2 expression and SOX-2 expressing cells in all cell cultures maintained in standard conditions. SOX-2 is a member of the SRY-related HMG-box (SOX) transcription factor family involved in several cellular processes, including maintenance of embryonic stem cells potency and differentiation of neural progenitor cells and cancer development [36–38]. SOX-2 knockdown has been reported to inhibit the sphere generation ability in glioma cell cultures as well the dedifferentiation and the stemness phenotype thus decreasing tumorigenicity [33–35, 39]. In particular, Gangemi *et al*. showed that GBM tumor-initiating cells would stop proliferating and lose tumorigenicity in immunosuppressant mice through silencing SOX-2 [34]. Yang *et al*. reported that the knockdown of the SOX-2 gene in the GBM cell line LN229 reduced cellular proliferation and colony formation [15]. Garros-Regulez *et al*. have reported that SOX2-SOX9 represents an oncogenic axis able to regulate stem cell properties and chemoresistance to TMZ [30]. More recently, Singh *et al*. showed that a set of three transcription factors, including SOX-2, represents an ultimate driver of glioblastoma [40]. Taken together, these data strongly suggested that SOX-2 is important for both tumorigenicity and drug resistance in glioblastoma stem cells.

To the extent of our knowledge, this is the first study that evaluates the relationship among NOS2 and SOX-2 levels in glioma primary cultures. The potential role of NOS2 as a key regulator in the aberrant upregulation of master genes such as SOX-2, involved in sustaining self-renewal of glioma initiating cells should be further investigated. In this context, in fact, the literature data are still scarce and involve different systems of stem cells. In particular, Kim *et al*. [29] found that irradiation of glioma cells promotes GSC through NOS2-mediated NO production. They reported that downregulation of NOS2 led to a reduced GSC population as well a decreased radiotherapy resistance by preventing NO generation. Of interest, siRNA targeting NOS2 blocked the increase in SOX2, Notch-2 and β-catenin expression in irradiated U87 glioma cells, thus inhibiting the stemness machinery for malignant progression. Moreover, the exposure of induced-pluripotent stem cells from mouse Sertoli cells to NO allowed them to maintain pluripotency through the activation of the pluripotent genes Oct4 and SOX-2 and upregulation of Nanog expression [41]. In this context, our findings could represent a useful contribution to the development of potential therapeutic approaches for the treatment of glioma based on knowledge of the signaling pathways involved in the NO-mediated glioma cell regulation. Studies are in progress to investigate the role of the glioma-associated inflammatory profile with the aim to identify the upstream mechanisms of NOS2 induction which in turn might underlie the overexpression of stemness markers such as SOX-2 and the consequent abnormal expansion of glioma initiating stem cells.

## MATERIALS AND METHODS

### Reagents

DMEM (Dulbecco's Modified Eagle Medium, high glucose), DMEM/F12 (1:1), Phosphate-buffered saline (PBS), Fetal Bovine Serum (FBS), Glutamine, Penicillin, Streptomycin and Trypan blue dye were obtained from EuroClone (Euro Clone, West York, UK). Trypsin from bovine pancreas (T1426), LPS (Lipopolysaccharides from Escherichia coli), Nitrate reductase, L-lactic dehydrogenase (LDH), pyruvic acid, b-nicotinamide adenine dinucleotide phosphate (NADPH), flavin adenine dinucleotide (FAD), HEPES, Griess reagent chemicals, and N-[[3-(aminomethyl) phenyl]methyl]-ethanimidamide dihydrochloride (1400W) were purchased from Sigma Chemical Co. (St. Louis, MO, USA). All cell cultures were seeded into a 100 mm sterile petri dishes and flasks acquired by EuroClone (Euro Clone, West York, UK). Optical microscope (Eclipse 50i) and inverted microscope (TS100) were purchased from Nikon (Nikon Corporation, Japan). 50X B-27® Serum-Free Supplement was acquired from Life Technology Corporation (CA, USA), EGF (Recombinant human Epidermal Growth Factor; 11343407), b-FGF (human basic Fibroblast Growth Factor) were acquired from ImmunoTools (26169 Friesoythe, Germany) and Accutase™ was from PAA-GE Healthcare Life Sciences (GE Healthcare Bio-Sciences AB, SE-751 84 Uppsala Sweden). To recover large size neurospheres, we used cell strainers 100μm nylon (352360, BD Falcon). IFN-γ, IL-1β, TNF-α were purchased from Cell Signaling Technology (Danvers, USA). For cytofluorimetric analysis, we used a FACSCalibur equipped with CellQuest software (Becton Dickinson, San Jose, CA). The following primary antibodies were used for immunostaining analysis of cell lines and primary cultures: anti-β-tubulin III (Alexa Fluor 488, 560338), anti-SOX-2 (PE Mouse, 560291), anti-nestin (Alexa Fluor 647 560393) all acquired from Becton Dickinson. All reagents for the reverse transcriptase reaction and Trizol Reagent were purchased from Invitrogen Life Technology (Invitrogen Corporation, CA, USA) and all PCR reagents and EuroSafe Nucleic Acid Staining Solution (20.000x) were acquired from EuroClone (Euro Clone, West York, UK). Primers were acquired from IDT (Integrated DNA Technologies, Coralville, USA). The RT-PCR reaction was made by thermocycler GeneAmp PCR System 9700 (Applied Biosystems CA, USA). PCR quantification was performed by densitometer (UVItec Limited BTS -20M, Cambridge UK) and densitometric analysis was made using ImageJ software.

### Cell lines

The human glioma cell lines T98G (grade IV glioma, GBM), U-87 MG (grade IV glioma, GBM), U-251 MG (grade IV glioma, GBM), U-373 MG (grade III astrocytoma), and LN229 (grade IV glioma, GBM) were obtained from ATCC (American Type Culture Collection, Georgetown, DC, USA). T98G, U-87 MG, U-251 MG and U-373 MG were cultured in DMEM supplemented with 10% of Fetal Bovine Serum (FBS), 100 U/ml penicillin, 100 mg/ml streptomycin and 2 mM glutamine (standard condition- “St-M”). LN229 were cultured in same medium DMEM supplemented with FBS 5%. All flasks were incubated in sterile conditions at 37°C in a humidified atmosphere with 5% CO_2_. After reaching 80% confluence adherent cell cultures were expanded; subculturing was performed every 3 days and the culture medium was totally replaced by centrifugation for 10 min at 400*xg*. Cell viability was evaluated using an exclusion assay based on trypan blue dye (0.04% PBS) and cells were visualized in a Bürker chamber and counted by microscopy.

For neurosphere generation (GSC), all glioma cell lines were cultured in DMEM/F12 (1:1, vol/vol) serum free, a specific medium for brain tumor stem cell growth, containing 20 ng/ml of both recombinant human epidermal growth factor (EGF) and fibroblast growth factor-basic (b-FGF) added with B27 supplement, penicillin/streptomycin and glutamine, as previously described [42] (“GSC-M”). When neurospheres grew in size (about 100 μm) were enzymatically dissociated with the Accutase™ solution for about 10 min at 37°C, ensuring a gentle and effective dissociation of cell aggregates and maintenance of surface proteins and epitopes. All cell lines in DMEM 10% FBS and respective neurospheres morphology were visualized and images were taken by Nikon Eclipse TS100.

### Ethical statement and human glioma samples

This study was ethically approved (Hospital Ethics Committee), and all patients affected by malignant glioma at several grade, as confirmed by neuropathological examination, underwent a surgical exeresis, in accordance with fluorescence-guided tumor resection protocol (ALA-PDD assisted resection). Each patient gave written informed consent. In this study, 10 glioma biopsies were obtained from Neurosurgery Unit, San Salvatore Hospital of L’Aquila. In particular 6 biopsies of IV grade, 1 of III grade and 3 of low grade glioma were processed in order to obtain primary and neurospheres cultures. Detailed clinicopathologic characteristics of all the patients are reported in Table 3.

**Table 3:** Clinical features of the patients enrolled in this study

Patient	Sex (M/F)	Age (years old)	Side of tumor	Date of surgery (year, month)	Histological diagnosis (WHO classification)	Months of survival	Preoperative KPS	Postoperative KPS	Preoperative Deficit	Postoperative Deficit (at the discarge)	Postoperative treatment	Recurrence
1	M	77	Right fronto-pariel	2011, Sept.	IV grade	6	70	70	Moderate left hemiparesis	No further deficit than preop time	CT + RT (Stupp protocol*), Levetiracetam	After 6 months
2	M	75	Right fronto-temporal	2013, Sept.	IV grade	11	80	80	Mild ideo-motor apraxia, mild left lower limb deficit with some difficulties with walking	No further deficit than preop time	CT + RT (Stupp protocol*), Levetiracetam	After 10 months
3	F	72	Right fronto-temporal	2013, Sept.	IV grade	11	80	90	Mild ideo-motor slowing, mild motor deficit in left upper limb	Improvement of ideo-motor slowing and preop motor deficit	CT + RT (Stupp protocol*), Levetiracetam	No
4	M	36	Left Temporal	2013, Sept.	III grade	Living	100	100	No deficit	No deficit	RT	No
5	F	45	Left frontal	2013, Oct.	II grade	Living	90	90	Dysphasia	Improvement of preoperative dysphasia	RT after the first operation. CT after the second operation. Levetiracetam	After 22 month (Re-operation)
6	F	48	Left frontal	2013, Dec.	II grade	Living	90	100	Dysphasia	Improvement of preoperative dysphasia	RT. Levetiracetam	No
7	F	65	Left fronto-temporal	2013, Nov.	IV grade	17	80	80	Right hemiparesis (Moderate in upper limb, mild in lower limb)	No further deficit than preop time	CT+ RT (Stupp protocol*). Levetiracetam	After 13 months
8	F	37	Right temporal	2014, Nov.	IV grade	Living	90	90	No deficit	No deficit	CT+ RT (Stupp protocol*). Levetiracetam	No
9	F	68	Left Temporo-parietal	2015, Oct.	IV grade	14	90	90	Mild ideo-motor slowing, very mild right upper limb deficit	No further deficit than preop time	RT + CT (Stupp protocol*). Levetiracetam	After 10 months
10	F	27	Left temporal	2016, Feb.	II grade	Living	100	100	Dysphasia	No deficit	RT + CT (Stupp protocol*). Levetiracetam	No

*Stupp protocol: Adjuvant treatment with radiotherapy (RT) and chemotherapy (CT). Focal radiotherapy was delivered once daily at 2Gy per fraction, 5 day/week, for a total of 60 Gy, as prescribed by the radiation oncologist, according to the guidelines of the International Commission on Radiological Units. Concomitant treatment with temozolomide was prescribed by the oncologist at a dose of 75 mg/m^2^ for each day of radiation treatment. Four weeks after radiotherapy, patients received adjuvant temozolomide chemotherapy, 150 to 200 mg/m^2^ on days 1 to 5 at 28-day intervals. All patients were subjected to radical exeresis, except patient #10 subjected to a subtotal exeresis.

### Glioma primary cultures and derived neurospheres

From each of 10 human solid gliomas, clinically and histologically characterized, we obtained primary cultures as previously described [9]. Briefly, fresh surgical specimens were washed in PBS in order to remove adhering blood and visible necrotic portions. Mechanical and enzymatic tissue dissociation by trypsin solution, was carried out in order to obtain single cell suspensions; biopsies digestion was performed at 37°C for 15-20 min in a water bath by gentle stirring. A rate of recovered cells was cultured in medium containing DMEM supplemented with 10% FBS, 100 U/ml penicillin, 100 mg/ml streptomycin and 2 mM glutamine (complete medium) and another rate was suspended in DMEM/F12 serum free medium supplemented with 20 mg/ml EGF, 20 mg/ml b-FGF and B27. *In vitro*, the generated neurospheres appeared typically as free-floating structures. All flasks were incubated in sterile conditions at 37°C in a 5% CO_2_ humidified atmosphere and the complete medium was totally replaced every three days. After reaching 80% confluence, glioma primary cultures were expanded and their morphology was acquired by Nikon Eclipse TS100 microscope. Cell viability was determined using the trypan blue dye, as previously described for glioma cell lines.

### Immunofluorescence analysis of stemness markers

Immunofluorescence analysis of glioma cell lines, glioma primary cultures and respective GSCs was performed by FACSCalibur flow cytometry in order to analyze stemness markers expression (β-tubulin III, SOX-2, and nestin). Tumor spheres were dissociated as single cells by Accutase™ solution and single cell suspensions were fixed for 15 min at room temperature with 2% formaldehyde in PBS. Cells were permeabilized for 5 min at room temperature with 0.1% Triton-X-100 in PBS and then washed twice. At least 2 × 10^5^ cells for each experimental condition were incubated with a primary fluorochrome-conjugated monoclonal antibody for 1 h at room temperature in the dark. Samples were centrifuged at 400*xg* for 10 min and washed twice. 10,000 events were visualized for each sample. The population of interest was gated according to its Forward Scatter (FSC)/Side Scatter (SSC) criteria. Data were analyzed using CellQuest software.

### Total RNA extraction and gene expression by reverse transcriptase PCR (RT-PCR)

Total RNA from all glioma specimens was extracted using Trizol Reagent according to the manufacturer's protocol. RNA for NOS2 positive control was obtained from immortalized human non-small cell lung cancer A549 cells (ATCC) treated with inflammatory cytokines (IFN-γ 10 ng/ml, IL-1β 10 ng/ml and TNF-α 10 ng/ml) and LPS 100 μg/ml.

RNA was spectrophotometrically quantified and its quality was assessed by 1% agarose/Tris–Acetate–EDTA (TAE) gel electrophoresis. The gene expressions were quantified in a reverse transcription-polymerase chain reaction (RT-PCR). To prepare first-strand cDNA, 1 μg of total RNA was reverse transcribed with reverse transcriptase enzyme MLV-Reverse Transcriptase in 20 μl reaction mixture. For the reverse transcription reaction a mixture of 20 μl including total RNA sample (1 μg), 0.5 μg/μl Oligo (dT) 12–18 primer and 10 mM of the four deoxynucleoside triphosphates (dNTPs) for each sample was used. Samples were incubated at 65°C for 5 min followed 37°C for 2 min. A reaction buffer containing 5X First Strand Buffer RT, 0.1 M dithiothreitol (DTT), 40 U/μl of a ribonuclease inhibitor was added to each sample. Following incubation at 37° C for 2 min, 200 U/μl of M-MLV Reverse Transcriptase was added in every sample. PCR products were synthesized from cDNA using specific primers for NOS2. A relative quantification method was employed where the mRNA level of a target gene was normalized with β-actin mRNA sequence (internal control) used as housekeeping gene. The oligonucleotide primers were designed from NOS2 sequence (GenBank accession number NM 153292) and from human cytoplasmic β-actin gene sequence (GenBank accession number M10277) as reported in Table 4. PCR step was carried out in a volume of 50 μl, including 10 μl of cDNA, 4 mM MgCl_2_, 10 mM of the dNTPs mix, buffer 10X, 5 U/μl of AmpliTaq DNA polymerase and 100 μM each of NOS2 and β-actin primers. The PCR products were analysed on 1.2% agarose gel and visualized by EuroSafe Nucleic Acid Staining. The PCR conditions for NOS2 and β-actin were: 35 cycles of denaturation at 94°C for 1 minute, annealing at 61°C for 1 min and extension at 72°C for 1 min. Densitometric analysis was performed using Image J Software to quantify the band intensities. Data represent average values from three independent experiments.

**Table 4:** Synthetic oligonucleotide primers used for RT-PCR analysis

RefSeq	PRIMER NAME and SEQUENCE	Product size (base pairs)
NM 153292	*NOS2 FOR5′-ctgacgggagatgagctc- 3′*	210 bp
	*NOS2 REV5′-agtcgtgcttgccatcactc- 3′*	
M10277	*β-actin FOR5′-agcgggaaatcgtgcgtg- 3′*	300 bp
	*β-actin REV5′-cagggtacatggtggtgcc- 3′*	

FOR, forward; REV, reverse.

### Nitrite/Nitrate level assay

Nitric oxide is difficult to quantitate because it is produced in small amounts under most conditions and has a short half-life, however, measuring the accumulation of nitrite and nitrate is a useful way to quantitate NOS activity [43]. The enzymatic activity of NOS2 was thus evaluated by measuring nitrite levels using nitrate reductase and Griess reaction through a colorimetric assay. Briefly, supernatants of cell cultures (100 μl) were applied to a microtiter plate well, followed by Hepes 50 mM, FAD 5 μM, NADPH 0.1 mM, nitrate reductase 0,2 U/ml, lactic dehydrogenase (LDH) 1500 U/ml and pyruvic acid 100 mM, and finally the Griess reagent. The absorbance was measured by spectrophotometric reading at 550 nm. The values were interpolated with a standard curve with known concentrations of KNO3.

### Statistical analysis

All data were analysed using Prism 5.0 GraphPad Software, San Diego, Ca. For comparison between two means, Student's unpaired *t* test was used. Statistical correlation was calculated through Pearson's test. Data were considered statistically significant at a *p* value less than 0.05.

The Authors thank Gasperina De Nuntiis (Department of Life, Health & Environmental Sciences, University of L’Aquila, L’Aquila, Italy) for technical assistance.

Paola Palumbo and Gianfranca Miconi: generation of primary cultures from glioma biopsies, cell culture maintaining, RT-PCR experiments, and data acquisition.

Benedetta Cinque: cytofluorimetry and immunofluorescence analyses.

Francesca Lombardi, and Cristina La Torre: analysis of nitrite levels and cell culture maintaining.

Paola Palumbo, Francesca Lombardi, and Cristina La Torre: statistical analysis.

Renato Galzio and Soheila Dehcordi Raysi: surgery and all clinical patients’ evaluation.

Paola Palumbo, Maria Grazia Cifone, AnnaMaria Cimini, and Antonio Giordano: supervision of development of work and data interpretation.

Paola Palumbo, Benedetta Cinque, and Maria Grazia Cifone: drafting the original version as well writing and editing the revised manuscript.

## SUPPLEMENTARY MATERIALS


