# Supplementary material for: NOS2 expression in glioma cell lines and glioma primary cell cultures: correlation with neurosphere generation and SOX-2 expression
Source: Oncotarget. 2017 Mar 10;8(15):25582–98. doi: 10.18632/oncotarget.16106 (PMC5421953; doi:10.18632/oncotarget.16106)
Supplement: Supplementary file 1 [file oncotarget-08-25582-s001.pdf]

NOS2 expression in glioma cell lines and glioma primary cell cultures: correlation with neurosphere generation and SOX-2 expression

SUPPLEMENTARY MATERIALS

Surgical excision, technical notes

Since gliomas are intrinsic tumors of the neocerebrum, there are visible intraoperative pathomorphological changes identifyng the tumor site. Generally, we recognize the involved gyrus because it is whitened or it appears swollen¸ moreover the sulcal veins may be visibly reddened, indicating shunted tumoral blood. Generally glioma stay subpial, but malignant lesions tend to break the pia mater and to be adherent to surrounding arteries and veins within the sulci and fissures. In the most of cases, we prefer microsurgically debulking glioma, beginning peripherally, to avoid dangerous bleeding. In our opinion, it's extremely important to remove the lesion *centripetally*, creating space along the circumferential tumor-brain interface. Inserting sponges could be useful in restoring a contour along the interface to aid the dissection.

In order to provide updated information as the surgical procedure progresses, we constantly make use of the Neuronavigation. This latter provide data regarding orientation during the entire surgical procedure, including details of structures adjacent or deep to the area of immediate dissection, as well as details about the lesion in question (such as the amount of remaining tumor, position of deeply located - encased or displaced - neural and vascular structures, etc.). It is a very useful tool in defining lesional limits and boundaries, facilitating radical and safer exeresis. In addition, for periventricular and deep-seated malignant glioma we usually use 5-aminolevulinic acid (ALA), which helps to have a complete removal of the mass, checking any residuals (ALA-PDD assisted resection).

Clinical considerations

The results of this study, albeit on a limited series of patients, should lead to clinicians to go beyond the clinical evaluations and account the biomolecular profile of each patient to identify a common features notwithstanding the patient variability.

Biomolecular results showed presence of GSCs in one case of low grade glioma (sample #10 of Figure 7A), with higher and significant NOS2 expression in primary cultures in St-M when compared to the respective neurospheres culture.

Analysing clinical data, we noted that the sample #10 corresponds to a patient (young girl of 27 years old) with a left temporal glioma who has undergone to a surgery of *subtotal* exeresis. In this case it was not possible a radical excision because the tumor was very extensive, deeply located and involving eloquent areas. It is likely that, although histological analysis has suggested a low grade glioma, the lesion has a malignant behaviour, as well as it is not excluded that the non-excised tumor is composed of malignant neoplastic cells. Therefore, in her postoperative time, the patient underwent adjuvant radio- and chemotherapy. She is still alive and she is subjected to a close clinical (i.e. neurological exam and laboratory tests of blood) and instrumental (i.e. Magnetic resonance imaging and electroencephalographic exams) follow-up. Moreover, no patient has come back to work in the postoperative time, except for patients #4, #5 and #6.
